# Review on Microwave-Matter Interaction Fundamentals and Efficient Microwave-Associated Heating Strategies

**DOI:** 10.3390/ma9040231

**Published:** 2016-03-25

**Authors:** Jing Sun, Wenlong Wang, Qinyan Yue

**Affiliations:** 1 School of Environmental Science and Engineering, Shandong University, Jinan 250100, China; sunjing0108@163.com; 2 National Engineering Laboratory for Coal-fired Pollutants Emission Reduction, School of Energy and Power Engineering, Shandong University, Jinan 250061, China

**Keywords:** microwave, mechanisms, dielectric heating, magnetic heating, discharge, efficiency

## Abstract

Microwave heating is rapidly emerging as an effective and efficient tool in various technological and scientific fields. A comprehensive understanding of the fundamentals of microwave–matter interactions is the precondition for better utilization of microwave technology. However, microwave heating is usually only known as dielectric heating, and the contribution of the magnetic field component of microwaves is often ignored, which, in fact, contributes greatly to microwave heating of some aqueous electrolyte solutions, magnetic dielectric materials and certain conductive powder materials, *etc.* This paper focuses on this point and presents a careful review of microwave heating mechanisms in a comprehensive manner. Moreover, in addition to the acknowledged conventional microwave heating mechanisms, the special interaction mechanisms between microwave and metal-based materials are attracting increasing interest for a variety of metallurgical, plasma and discharge applications, and therefore are reviewed particularly regarding the aspects of the reflection, heating and discharge effects. Finally, several distinct strategies to improve microwave energy utilization efficiencies are proposed and discussed with the aim of tackling the energy-efficiency-related issues arising from the application of microwave heating. This work can present a strategic guideline for the developed understanding and utilization of the microwave heating technology.

## 1. Introduction

Microwaves are part of the electromagnetic spectrum that move at the speed of light with a wavelength ranging from 1 m to 1 mm, which corresponds to a frequency range of 300 MHz to 300 GHz. Different from the conventional heating, microwave heating involves an energy conversion from the electromagnetic energy to thermal energy rather than the heat transfer. Microwave energy is delivered directly to the material through molecular interaction with the electromagnetic field. Since microwaves can penetrate the material and supply energy, heat can be generated throughout the volume of the material resulting in volumetric heating [1,2,3,4,5,6,7,8,9,10,11]. Besides volumetric heating, microwave heating offers a number of advantages such as: (1) selective material heating; (2) rapid heating; (3) non-contact heating; (4) quick start-up and stopping; (5) the ability to treat waste *in-situ*; (6) and portability of equipment and processes [12,13]. Based on these distinguished advantages, microwaves have been used in various technological and scientific fields, for example: food processing; different drying processes; as a remedial tool used in the area of sludge processing, medical waste treatment, contaminated soil remediation, waste water cleanup, and activated carbon regeneration; sintering of metals andceramics; plasma processing; preparation of functional materials; pollution control; pyrolysis processes and many other physical and chemical fields in recent years [4,5,8,10,11,14,15,16,17,18,19,20,21,22,23,24].

Microwave heating is often known as dielectric heating. Dielectric heating refers to heating by the electric field (E-field) component of the high-frequency electromagnetic radiation, owing to the presence of electric dipoles in polar molecules. For example, the microwave heating of water is dielectric heating owing to the dipolar polarization. In addition to the E-field, microwaves also have a magnetic field (H-field) component which also couples with some materials to induce heating. Consequently, in addition to the dielectric heating, there are other types of microwave heating such as magnetic loss heating, Joule heating caused by conductive losses, *etc.* However, the extended understanding of the microwave-matter interaction mechanisms other than the dielectric heating is still very insufficient rather than popular. If comprehensive insight into these mechanisms is well developed, interesting opportunities may open for better and extended use of microwave heating in more fields.

In addition to the acknowledged conventional microwave heating mechanisms, as a special type of material, the interaction mechanisms become more complicated when metals and their alloys are subjected to a microwave field. It is well known that bulk metals do not couple directly with microwave energy but readily reflect the incident waves. However, metal particles can be heated in a microwave field when they are present in a sufficiently small size [25]. For instance, microwave heating has been successfully applied to the sintering of powdered metals and alloys in the past two decades [18,26,27,28,29,30,31,32,33,34,35,36,37]. Despite the reflection effect and heating effect, microwave-induced electric discharges could also occur in the forms of corona, arc, spark and/or some weak discharges when metals with sharp edges, tips and submicroscopic irregularities are subjected to a microwave field since the charges on the metal surface accumulate at these macro-, micro- or submicroscopic irregularities and will jump out of the material when they accumulate enough kinetic energy, resulting in the ionization and breakdown of the surrounding medium. As a direct consequence, considerable heat is produced during the discharge process, leading to the formation of high temperature local hotspot since the discharge process is transient and concentrated. At a microscopic level, these hotspots are actually plasmas [12]. An intensive generation of such discharge/plasmas may have significant implications for a variety of processes involved [38,39]. Consequently, the special effects caused by microwave-metal interaction cannot be ignored and may play an important role in various fields. It is of great importance to disclose the special interaction mechanisms and associated potential effects between microwave and metallic materials.

This paper is aimed to present a strategic guideline for better utilization of microwave technology through reviewing microwave interaction mechanisms with different classes of materials. The detailed mechanisms of microwave E-field heating and H-field heating are reviewed firstly in Section 2. As microwave heating is increasingly used in a variety of metallurgical, plasma and discharge processes, the special interaction mechanisms between microwave and metallic materials are summarized in Section 3. Since energy efficiency is an important issue for the practical applications of microwave technology, the strategies to enhance the microwave energy efficiency are of growing importance and are discussed in Section 4. Finally, directions for further research and potential applications are suggested.

## 2. Microwave Heating Theory and Mechanisms

When electromagnetic waves encounter a medium, the waves can be reflected, absorbed, transmitted or any combination of these three interactions. There are many mechanisms responsible for the microwaves-matter interactions, which can be generalized as dielectric losses, conductive losses, and magnetic losses, *etc.* These mechanisms are responsible for the commonly known forms of heating such as dielectric, Joule, and induction heating, all of which are dependent on the electromagnetic field characteristics and the material’s properties [40,41]. As the electromagnetic field has two components, the electric field component and the magnetic field component, the different components will interact with materials according to different mechanisms. A detailed discussion of their respective mechanisms is of great significance to the development of microwave heating fundamentals and applications.

### 2.1. Microwave Electric Field Heating

The electric field component of microwaves is responsible for the dielectric heating. In the frequency range of microwaves, the dielectric heating is effected via two primary mechanisms, dipolar polarization and ionic conduction. In polarization mechanism, a dipole is sensitive to external electric fields and will attempt to align itself with the field by rotation. Under a high frequency electric field, the dipoles do not have sufficient time to respond to the oscillating field, as a result of this phase lag, they collide with eachother when they attempt to follow the field and power is dissipated to generate heat in the material. This is the case for water and other polar materials. The dipolar polarization mechanism is the primary principle of microwave dielectric heating that involves the heating of electrically insulating materials by dielectric loss. In conduction mechanism, any mobile charge carriers (electrons, ions, *etc.*) move back and forth through the material under the influence of the microwave E-field, creating an electric current. These induced currents will cause heating in the sample due to any electrical resistance caused by the collisions of charged species with neighboring molecules or atoms.

In certain situations, these two mechanisms can not be separated from each other and work together in microwave heating which is very necessary for such a heating system that includes a conducting material scattered in a non-conducting medium (e.g., electrolyte solutions). It was reported that heating rates of the electrolyte solutions (NaCl, KCl, CaCl_2_, NaBF_4_, and NaBr) were far more significant than that of same-volume ultrapure water when they are heated in the same microwave conditions [42]. For the aqueous electrolyte solutions, both dipolar polarization and conductive mechanisms contribute to the heating effect.

Despite of the aqueous electrolyte solutions, in the case of dielectric solid materials with charged particles which are free to move in a delimited region of the material, such as π-electrons in carbon materials, a current traveling in phase with the electromagnetic field is also induced. As the electrons cannot couple to the changes of the phase of the electric field, energy is dissipated in the form of heat due to the so-called Interfacial or Maxwell-Wagner Polarization effect [12].

As is well known, the power loss per unit volume for dielectric heating is governed by [43]:

(1) $$P=\omega \cdot \varepsilon_{eff}^{\prime \prime }\cdot \varepsilon_{0}\cdot E_{rms}^{2}$$

where *P* represents the power density in the material (W/m^3^) at the position (x, y, z); 𝜔 = 2𝜋𝑓 (Hz); *f* is frequency of the incident microwaves; $\varepsilon_{eff}^{\prime \prime }$ is the effective dielectric loss factor; $\varepsilon_{0}$ is the permittivity of free space ($\varepsilon_{0}$ = 8.854 × 10^−12^ F/m); $E_{rms}$ represents the local (at the position x, y, z) value of the electric field strength (V/m). With the consideration of the dipolar polarization, ionic conduction and Maxwell-Wagner polarization mechanisms, the effective dielectric loss factor ($\varepsilon_{eff}^{\prime \prime })$ for the dielectric heating expression (Equation (1)) can be expressed in a comprehensive manner, as:

(2) $$\varepsilon_{eff}^{\prime \prime }=\varepsilon_{Polarization}^{\prime \prime }+\varepsilon_{conduction}^{\prime \prime }=\varepsilon_{dipolar}^{\prime \prime }+\varepsilon_{interfacial}^{\prime \prime }+\sigma /\omega \varepsilon_{0}$$

### 2.2. Microwave Magnetic Field Heating

Compared with the electric field heating, there are not a large number of papers ascribing the microwave heating effect to the magnetic field (H-field) component. However, the superior advantages of microwave magnetic field heating over electric field heating for some materials has received increasing attention [44]. Cheng *et al.* first published their founding that microwave magnetic field was more efficient than the electric field for heating some magnetic dielectric materials (e.g., ferrite) and certain conductive powder materials [45,46,47]. The similar results and observations were then reported and confirmed by other researchers [44,48,49]. Zhiwei Peng reported the magnetic loss can be up to about four times greater than the dielectric loss in the microwave heating of ferrites (BaFe_12_O_19_, SrFe_12_O_19_, *etc.*) at 2.45 GHz [44]. Rosa *et al.* [50] also reported microwave heating of ferromagnetic powders presents a strong contribution by the H field interaction with matter, which, in regions of predominant magnetic field, can result significantly higher than the electric field related contribution. Apparently, magnetic field also contributes greatly to microwave heating which should not be ignored any longer. In fact, the magnetic loss contributes significantly to the microwave heating of a broad range of materials including magnetic materials, conductors, semiconductor materials and some others. Microwave magnetic field heating generates a distinct difference from the dielectric heating owing to the microwave electric field component, albeit still to be understood. To date, the principal mechanisms for microwave H-field heating are eddy current losses, hysteresis losses, magnetic resonance losses (including domain wall resonance and electron spin resonance), and residual losses.

Eddy current loss is the Joule loss due to the eddy current induced by the alternating magnetic field. Eddy currents can be generated whenever relative motion between a conductive material and an external magnetic field exists [51,52]. Generation of eddy currents during microwave processing of metals is caused when a conductor is exposed to a changing magnetic field due to relative motion of the field source and conductor, or due to variations of the field with time [51,52]. The eddy current density can be expressed as *J = σE*, where σ is electric conductivity, and ***E*** is the electric field induced by the alternating *H* field. Thus, from the view of Ohmic heating induced by an alternating magnetic field, the eddy current loss depends much on the electrical resistivity of the material. The eddy current loss is the primary mechanism for microwave heating of a broad range of conductor and semiconductor materials owing to the substantial contributions of the magnetic field [46,53,54].

**Figure 1:**
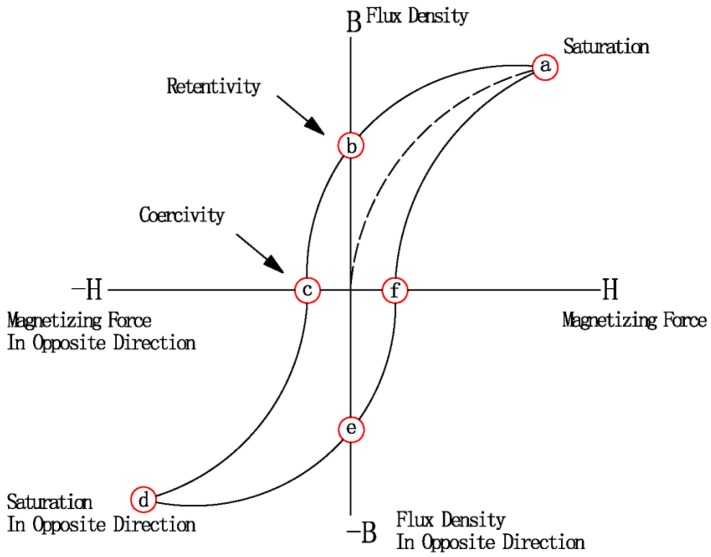
Typical magnetization (*B vs. H*) curve.

Hysteresis loss is caused by the irreversible magnetization process in the alternating magnetic field [55]. Thus, hysteresis losses occur only in magnetic materials such as ferrous material, steel, nickel, and a few other metals. When magnetic materials are subjected to an alternating magnetic field, the magnetic dipoles will oscillate as the magnetic poles change their polar orientation every cycle [56]. This rapid flipping of the magnetic domains causes considerable friction and heating inside the material. Heating due to this oscillation mechanism is known as hysteresis loss. When an alternating magnetic field is exerted on the material, its magnetization will trace out a loop called hysteresis loop [56]. The energy loss per circle is the area within the hysteresis loop by plotting the *B versus H* hysteresis curve for the material [25]. Figure 1 shows a typical magnetization (*B vs. H*) curve. Hysteresis loss contributes a lot to the heating of ferrous magnetic materials in an alternating magnetic field. Also, it should be pointed out steel will lose its magnetic properties to become nonmagnetic when heated above the Curie temperature (approximately 700 °C), and hysteresis ceases. This means that there can be no heating of the material due to hysteresis losses once upon above the Curie temperature.

Despite the eddy current and hysteresis losses, magnetic resonance losses also contribute a lot to microwave heating of some metal oxides such as ferrites and other magnetic materials. Magnetic resonance losses are primarily induced by domain wall resonance and electron spin resonance [42,46,57]. In the literature, residual losses were also used to represent the losses originating from various magnetic relaxations and resonances which occurs mainly in two ways, as rotational resonance and as domain wall resonance [55,58]. Accordingly, the magnetic resonance loss can be regarded as the residual loss or its components.

Conclusively, the eddy current loss is a large contributing factor for microwave heating of a broad range of conductor and semiconductor materials. Hysteresis loss takes place inside ferrous magnetic materials. Magnetic resonance loss/residual loss contributes to the induction heating of ferrite and other magnetic materials in an alternating magnetic field. In some situations, the eddy current loss, hysteresis loss and residual loss work together to contribute to the heating of some conductive magnetic materials (e.g., ferrite materials) in an alternating magnetic field.

In an analogous way to the dielectric losses in the electronic field, the power dissipated per unit volume into a material in the magnetic field (expressed in W/m^3^) can be described as [43]:

(3) $$P=\omega \cdot \mu_{eff}^{\prime \prime }\cdot \mu_{0}\cdot H_{rms}^{2}$$

where $\mu_{0}$ is the vacuum magnetic permeability ($\mu_{0}=4\pi \times 10^{-7}H/m$); $H_{rms}$ represents the local (at the position x, y, z) value of the magnetic field strength (A/m); and $\mu_{eff}^{\prime \prime }$ describes the imaginary part of the effective magnetic permeability which can be expressed in terms of three main contributors (hysteresis loss, eddy current loss and residual loss), as follows:

(4) $$\mu_{eff}^{\prime \prime }=\mu_{hysteresis}^{\prime \prime }+\mu_{eddy\;current}^{\prime \prime }+\mu_{residual}^{\prime \prime }$$

### 2.3. Power Loss Owing to Microwave Heating

Microwave heating is a process that involves direct energy conversion within the treated material since microwave can couple directly with the material to cause heat generation. The power dissipated per unit volume into the material can be described in a comprehensive manner as [43]:

(5) $$P=\omega \left(\mu_{0}\mu_{eff}^{\prime \prime }H_{rms}^{2}+\varepsilon_{0}\varepsilon_{eff}^{\prime \prime }E_{rms}^{2}\right)$$

This equation describes the power dissipated in the sample due to the magnetic field (1st term), the electric field (2nd term).

### 2.4. Penetration Depth of Microwaves

Even though the dielectric loss of a substance is fairly high, the heating efficiency for a large-size sample is sometimes low. This may be caused by the shallow penetration depth of the microwaves into the heated sample. The penetration depth is a useful parameter to quantify the heating efficiency and uniformity of a sample by microwaves.

Penetration depth, *d*, is a measure of the depth of microwave penetration in a material which is defined as the distance from the surface to the place at which the magnitude of the field strength drops to e^−1^ (=0.368) of its value at the surface. There is also a definition of power penetration depth, *Dp*, which is defined as the distance at which the traveling wave power density reduces to e^−1^ of its value at the surface and is half the value of the field penetration depth, *d*.

The penetration depth, *d*, can be expressed in a general manner as [59,60]:

(6) $$d=2\times Dp=\frac{1}{\alpha }=\frac{\lambda_{0}}{\sqrt{2}\pi }{\left\{\varepsilon_{r}^{\prime \prime }\mu_{r}^{\prime \prime }-\varepsilon_{r}^{\prime }\mu_{r}^{\prime }+{\left[{\left(\varepsilon_{r}^{\prime }\mu_{r}^{\prime }\right)}^{2}+{\left(\varepsilon_{r}^{\prime \prime }\mu_{r}^{\prime \prime }\right)}^{2}+{\left(\varepsilon_{r}^{\prime }\mu_{r}^{\prime \prime }\right)}^{2}+{\left(\varepsilon_{r}^{\prime \prime }\mu_{r}^{\prime }\right)}^{2}\right]}^{1/2}\right\}}^{-1/2}$$

In terms of loss tangent, Equation (6) can be expressed as:

(7) $$d=2\times Dp=\frac{\sqrt{2}c}{\omega \sqrt{\varepsilon_{r}^{\prime }\mu_{r}^{\prime }}}{\left[{\left(1+tan^{2}\delta_{\varepsilon }tan^{2}\delta_{\mu }+tan^{2}\delta_{\mu }+tan^{2}\delta_{\varepsilon }\right)}^{1/2}+tan\delta_{\varepsilon }tan\delta_{\mu }-1\right]}^{-1/2}$$

where $\lambda_{0}$ is the wavelength in the free space, $\lambda_{0}=c/f$, *c* is the velocity of light, with free space velocity, $c=\sqrt{\varepsilon_{0}\mu_{0}}$; $\varepsilon_{r}^{\prime }$ is the relative dielectric constant which is a measure of the ability of the dielectric material to store electrical energy; $\varepsilon_{r}^{\prime \prime }$ is the relative dielectric loss which is a measure of the ability of a dielectric material to convert electromagnetic field energy into heat; $\mu_{r}^{\prime }$ is the relative magnetic constant which is a measure of the ability of the material to store magnetic energy; $\mu_{r}^{\prime \prime }$ is the relative magnetic loss factor which represents the loss of magnetic field energy; $\tan\delta_{\varepsilon }$ is the dielectric loss tangent, $\tan\delta_{\varepsilon }=\varepsilon_{r}^{\prime \prime }/\varepsilon_{r}^{\prime }$; and $\tan\delta_{\mu }$ is the magnetic loss tangent, $\tan\delta_{\mu }=\mu_{r}^{\prime \prime }/\mu_{r}^{\prime }$.

Obviously, the penetration depth decreases with the increase of the frequency and the effective dielectric and magnetic loss factor. In other words, when the frequency is set, a material that has a high capability to convert the microwave energy to heat tends to have a low penetration depth. On the contrary, low-loss materials have a relatively large penetration depth, and in extreme conditions, some materials with extremely low loss factors (*i.e.*, high-purity quartz glass, and some non-plolar materials such as bezene and CCl_4_) can be almost transparent to microwaves, and therefore the penetration depth is very large and microwaves can pass through these materials with few energy loss. On the other side, when the penetration depth is much smaller than the sample dimension, penetration of microwave energy will be limited, thus making uniform heating impossible. The processing substance can be heated effectively by microwave when the penetration depths are correspondingly comparable to the sample dimensions.

Neglecting magnetic effects (*i.e.*, $\mu_{r}^{\prime \prime }=0,\;\;\mu^{\prime }=1$), the penetration depth can be expressed as [59,60,61]:

(8) d=2cω[ε′(1+(tanδε)2−1)]12

The penetration depth (also known as skin depth) of the magnetic field for a conductor which is a high loss medium can be expressed as [25]:

(9) $$d=\frac{1}{\alpha }=\sqrt{\frac{2}{\omega^{2}\mu_{0}\mu^{\prime }\varepsilon_{eff}^{\prime \prime }\varepsilon_{0}}}$$

When the polarization loss factor of the conductor is zero ($\varepsilon_{\mathrm{polarization}}^{\prime \prime }=0$), Equation (9) can be reduced to Equations (10) and (11):

(10) $$\sigma =\omega \varepsilon_{eff}^{\prime \prime }\varepsilon_{0}=1/\rho$$

(11) $$d=\frac{1}{\alpha }=\sqrt{\frac{2\rho }{\omega \mu_{0}\mu^{\prime }}}$$

where ρ refers to the resistivity of the material. For a perfect conductor, resistivity is zero, and therefore the penetration depth is zero. However, in most metals, total reflection of microwaves is impossible because of the limited resistance in the material due to the presence of defects. In general, the penetration depths for a variety of metals are limited to a few microns at the frequency range of microwaves, as is shown in Table 1.

In conclusion, the full consideration of the microwave penetration depth into a material is of great significance to an efficient and uniform microwave heating process. The optimal sample dimension should be comparable to the penetration depth, so that the entire sample can be bathed with microwaves to get the maximal heating rate.

## 3. Effects of Microwave-Metal Interactions

As microwave processing of metal-based materials is rapidly emerging as a fast and energy-efficient tool in powder metallurgy, catalytic pyrolysis, and some other fields, insight into the interaction mechanisms related to microwave and metallic materials is nowadays of great significance and hence gaining increasing attention from the scientific and technological community. The mechanisms are complicated because the different features of metallic materials may result in different performances under microwave irradiation. Even for the same kind of metallic material under the same microwave irradiation conditions, different performance levels will be obtained when the samples are of different structure or size. Basically, three featured effects will occur when metal-based materials are subjected to a microwave field. They are reflection effect, heating effect and discharge effect. We give a detailed explanation of these effects below.

### 3.1. Reflection Effect

In general, bulk metals do not couple directly with microwave energy but readily reflect the incident waves, allowing only surface penetration as no internal electrical field is induced in them. Although bulk metals cannot be heated effectively by microwaves, constructive and destructive interferences caused by the overlap of the traveling waves and reflection waves could alter the spatial distribution of the electromagnetic field intensity, resulting in changing the spatial power absorption patterns within the surrounding medium. Constructive interference leads to resonance and enhances power absorption, while destructive interference suppresses power absorption. A considerable amount of research has been carried out to investigate the role of metallic support/coating on the interference of the heating strategies [9,61,63,64,65,66,67]. For instance, Jolly and Turner carried out preliminary studies on microwave heating with reflective supports to control the temperature within 1D slab [66]. Basak and Priya [63] carried out the resonance studies of 1D slabs with a metallic support at the unexposed face; observed greater intensity of resonances for power absorption in samples (oil and water) occurs in presence of metallic support. They also proposed an efficient heating strategy with a composite support (metallic-ceramic) attached at the unexposed face [63].

Later, Basak [61,65] carried out studies on the spatial power absorption patterns within 2D samples with metallic plates or supports of various shapes to explore to how to focus microwaves efficiently to achieve larger power absorption within the sample through the application of foreign (metallic) support. They found various shapes of metallic annuli play a dominant role either to focus or to optimize heating effects, and the metallic annuli can enhance power absorption or heating rate for both bread and beef samples with specific aspect ratios (defined as the ratio of the dimension of metallic annulus and outer radius of the food sample) [65].

Additionally, Bhattacharya and Basak carried out investigations to find correlations of various limits of sample thicknesses and occurrence of resonance of spatial and average power absorption. Also, the suitable relationships between occurrence of resonance with sample size and reactor configuration (with or without foreign support) were established corresponding to a given material [68,69,70].

It should be pointed out that most work mentioned above is based on theoretical analysis via numerical modeling and a single mode microwave propagation, where the authors simplified the numerical calculation by assuming the heated sample were exposed to plane waves. The so-called single-mode cavity support only one mode and generally allow for a higher electromagnetic field strength and a well-defined spatial configuration of the electric and magnetic field; thus, single-mode cavities possess predictable field patterns and therefore a desirable heating profile can be devised. It is relatively easy to conduct theoretical analysis on the interference of the heating strategies owing to the introduction of foreign support in single-mode microwave propagation. However, as microwave cavities are broadly classified as either single-mode or multimode, despite the single-mode cavity, multimode cavities are also, or even more popular in the domestic and industrial applications. There is a great amount of experiments were performed in multimode—sometimes modified—household microwave ovens. The multimode cavity is more complicated than the single-mode one in essence. Multimode applicators are large cavities with all the dimensions much larger than the incident wavelength. This resonant cavity can host a large number of different spatial electromagnetic field configurations (modes). As a consequence of this, the overall electromagnetic field configuration in a multimode cavity can be seen as the resultant of the superimposition of different electromagnetic waves propagating in different directions due to the multiple reflections from the cavity walls [43]. Consequently, the role of metallic support/coating on the interference of the heating strategies in a multi-mode cavity should be more complicated than that in a single-mode cavity. To the best of authors’ knowledge, however, direct investigations on this issue are still very insufficient in the microwave heating technology literature, even though this topic would be of doubtless scientific interest. Conclusively, the suitable adoption of the metal support based on the material dielectric properties and sample dimensions to form the constructive resonance may open an important way to achieve an efficient microwave thermal process.

### 3.2. Heating Effect

Although the temperature of a bulk metal cannot be raised very much since it readily reflects the incident microwaves with small (order of micron or less) penetration depth of the electromagnetic field, the electromagnetic field does allow for the loosely bounded electrons to move and concentrate at surfaces, edges and points [71]. This results in two possible consequences; one is the discharge of the energy in the form of arcing [71] which will be discussed in the next section; the other is the heating effect since the induced eddy current on the metal surface is responsible for the Joule heating, and for ferro-magnetic metals, magnetic mechanisms are also considered to be responsible for the heating effect even though the detailed mechanisms of the magnetic loss in metals at GHz range are not fully understood [71,72,73]. For microwave heating of metallic materials, the penetration depth (also known as skin depth) of microwaves at a given frequency is a very important parameter since it constitutes an upper limit to the thickness of the material which can be heated directly by microwaves. According to Equation (11), the skin depth depends on the electrical and magnetic properties ($\mu_{r}^{\prime }$ and σ) of the material [74]. Materials with high conductivity and permeability present a lower penetration depth for a given frequency, but there is also implicit temperature dependence due to the changes of $\mu_{r}^{\prime }$ and σ [74]. In general, the skin depth is relatively small in metals, usually having a depth in the order of microns, so the direct heating tends to remain superficial; however, when the metal powders or particles have equivalent dimensions to the penetration depth, the surface area and thereby the "effective skin" (portion of metal powder that couples with microwaves) is high enough to contribute to its heating, and therefore, it is possible that some of the micron, submicron and nanosized metal powders undergo volumetric heating when subjected to microwaves [3]. Thus, in the case of metal powders or particles, microwaves can couple very efficiently with them when they are present in a sufficiently small size [25,71].

**Figure 2:**
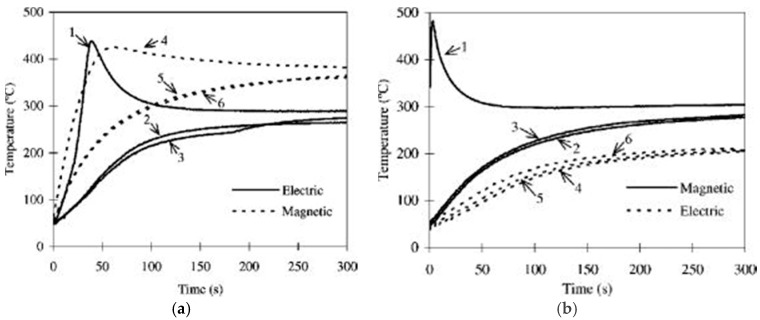
The heating behavior of the copper powder compact: first three times in E-field and then three times in H-field (**a**) *vs.* first three times in H-field and then three times in E-field (**b**) [76]. Reprinted from (J. Appl. Phys. 2007, 101, 074906) with the permission of AIP Publishing.

Microwave processing of metals and alloys have been robustly developed since the first successful literature on microwave processing of metallic powders provided by Roy *et al.* [28] in 1999. In that paper, the authors reported for the first time powdered metal components of various alloy compositions, including iron, steel, copper, aluminium, nickel, molybdenum, cobalt and tungsten, can be sintered in a multimode cavity at 2.45 GHz frequency and fully dense products with enhanced mechanical properties with respect to conventionally sintered counterparts were obtained in all cases. In subsequent articles, they observed that certain conducting materials heated well in the E-field, others in the H-field, and some like copper heated similarly in both fields [46]. They also reported various physical changes in materials, which they linked to differences in the interaction of the matter with the E- *vs.* H-field [36]. Luo *et al.* [75] studied the heating rate of pure iron and iron-nickel alloy powder compacts as a function of sample conductivity and frequency, and demonstrated magnetic induction heating best describes the loss mechanism in microwave heating of these kinds of powdered metals. Recently, Ma *et al.* [76] conducted a systematic study on the absorption, heating behavior, and microstructure evolution of porous copper powder metal compacts subjected to separated E- and H- field of 2.45 GHz microwave radiation, and found the microwave heating behavior of porous copper metal compacts depends significantly on whether the sample is initially subjected to the E- or H-field, the particle size, and (trivially) on the relative density of the compacts. Overall, compacts made from the same powder and same relative density heat more rapidly and to higher equilibrium temperature in the H-field compared to the E-field, as shown in Figure 2. They also found microwave heating can induce rapid changes in the absorption variables (*σ,* $\varepsilon^{\prime \prime }$, $\mu^{\prime \prime }$). More recently, Zhang and co-authors [77] reported a metal-semiconductor Fe/TiO_2_ composite exhibits a nonlinear dielectric resonance and multiple magnetic resonances, and found Fe grain size has great impact on the dielectric resonance which is attributed to interfacial polarization; the multiple magnetic resonances involve natural resonance and exchange resonances and the latter are dependent on the ferromagnetic grain size. Rosa *et al.* [50] demonstrated that in the presence of ferromagnetic powders loads, not only the power density, but also the extension of the regions where heat generation occurs are affected, depending on the load electrical and magnetic properties as well as on its arrangement inside the microwave cavity.

To date, microwave processing has gained worldwide acceptance as a novel method for heating and sintering a variety of metals and alloys since it offers many advantages in terms of enhanced diffusion processes, reduced energy consumption and processing cost, very rapid heating rates and significantly reduced processing times, improved physical and mechanical properties, simplicity, unique properties, new materials and products and lower environmental hazards, *etc.* [74,78,79,80,81,82]. For instance, Mondal *et al.* [31,32] reported that microwave sintering requires about 80% less processing time than conventional method and still provides better physical and mechanical properties. Zhou *et al.* [30] also pointed out the processing time required by microwave sintering can be 75% less than that required by conventional method and optimized heating rate leads to the enhanced mechanical properties of W and its alloys.

**Figure 3:**
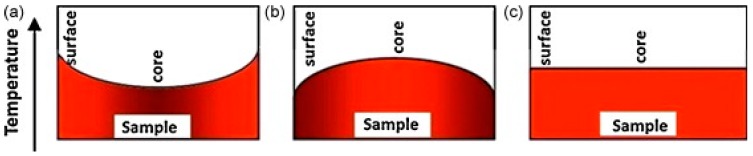
Temperature profile within the sample in: (**a**) conventional heating; (**b**) microwave heating; and (**c**) microwave hybrid heating [74]. Reprinted from (Journal of Alloys and Compounds, 2010, 494, 175-189) with permission from Elsevier.

Despite the distinguished advantages, the direct heating of materials with microwaves can encounter the fundamental problem of thermal instabilities which can cause temperature runaway into the processed materials. Moreover, some materials may not couple microwave power efficiently at room temperature and poor microwave absorption characteristics make initial heating difficult [83]. Since the fundamental difference between microwave sintering and conventional sintering is in the heating mechanism illustrated by the temperature profile in Figure 3, to overcome these problems, a hybrid heating technique, which is a combined action of microwaves and a microwave-coupled external heating source, was developed to realize rapid sintering from both inside and outside of the powder compact [84,85,86]. Materials having higher dielectric losses at room temperatures are used as an infrared heating source and are called as susceptor. The hybrid heating system will heat the sample more readily at low temperatures and at high temperatures will flatten out the temperature profile inside the sample body as is shown in Figure 3 [74,87]. Chandrasekaran *et al.* [85] conducted microwave heating and melting of lead, tin, aluminium and copper with the aid of susceptor and compared the melting characteristics of metals using the microwave of 1300W capacity and a muffle furnace (conventional) of 2500 W capacity. For all the metal used, compared to conventional melting, microwave melting was twice as fast and more energy efficient.

Aside from being already become a well-established and ordinary tool in metal sintering and melting, microwave heating has been innovatively applied and is nowadays gaining increasing popularity also in material processing. The typical applications are the ignition of combustion synthesis (CS)/self-propagating high-temperature synthesis (SHS). Combustion synthesis is widely recognized as an intriguing material processing technique with dramatic reduction in the energy consumption compared with conventional procedures since the energy input is limited to the ignition step, and after ignition, the exothermic reaction is able to start reacting in a self-sustaining regime as a result of the heat produced by the reaction itself. Thus, the only energy requirement is limited to the ignition step [43,50]. Among the large number of possible ignition techniques, microwave energy surely represents an attractive alternative since direct interaction of the microwave energy with absorbing reactive samples allows microwave-assisted ignition to have several advantages compared with the conventional thermal ignition including: faster and possibly volumetric energy transfer arising from microwave-material interaction, as well as the consequent savings of ignition time and energy, more localized ignition, a non-contact transfer of energy with contamination avoided, *etc.* [43,50]. Just owing to these distinct advantages, microwave ignition of the CS/SHS has been extensively studied in the past decade [43,50,88,89,90,91,92]. Naplocha and Granat [89,91,92] conducted microwave-activated combustion synthesis for manufacturing porous preforms of Al–Cr compounds and reported composite materials with significant hardness and oxidation resistance at high temperatures were produced. Rosa *et al.* [90]conducted combustion synthesis of γ-TiAl based alloys and reported β-NiAl-coated γ-TiAl-based alloys were synthesised following a single combustion synthesis step in a mono-mode microwave applicator operating at 2.45 GHz under a moderate pressure of 0.15 MPa, allowing the simultaneous synthesis and forming of the required shape. More recently, in one of their published papers, they investigated the effects of microwave ignition of CS reactions in regions of predominant electric (E) or magnetic (H) field formixtures of aluminium powders with powders ofone ferromagnetic metal reactant (namely Fe, Co and Ni), in order to propose new strategies to optimize the synthesis of high purity aluminide intermetallics, and found the ignition of the combustion reactions in predominant magnetic field allowed to significantly reduce the ignition times, with a global reduction of the power required to synthesize the intermetallics, increase the reproducibility of the experiments and the possibility to avoid electric arcs generation [50]. In another their recently published paper [93], the rapid microwave heating of metallic powders mixtures compacts was applied to produce Si-modified Mn_25_FexNi_25_Cu_(50-x)_, (x = 25, 30, 35, 40) high entropy alloys. With the further understanding of microwave-matter interaction mechanisms, the advantages of microwave heating will be doubtlessly exploited and applied in more fields.

### 3.3. Discharge Effect

**Figure 4:**
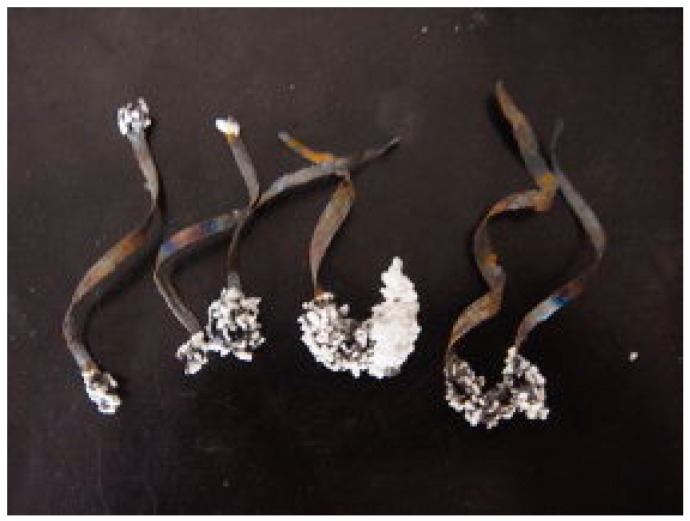
Appearance of stainless-steel strips after microwave discharge [96]. Reprinted from (AIChE Journal, 2012, 58, 3852–3857) with permission from John Wiley and Sons.

**Figure 5:**
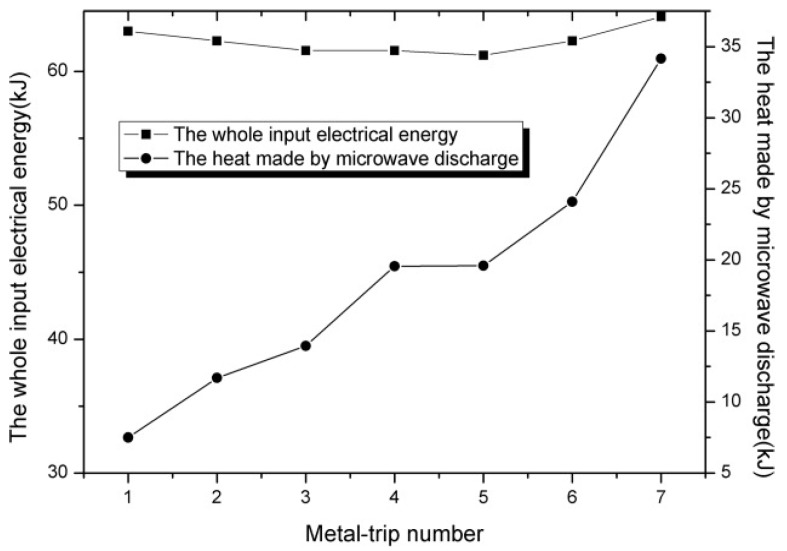
The electric energy comsumed and heat generated by microwave-metal discharge in relation to metal-strip number [96]. Reprinted from (AIChE Journal, 2012, 58, 3852–3857) with permission from John Wiley and Sons.

Despite the reflection effect and heating effect mentioned above, microwave fields also alter the distribution of the positive and negative charges on the conducting material and an additional unique phenomenon may take place when metals with sharp edges, tips or submicroscopic irregularities are subject to microwaves irradiation. This phenomenon is usually an electric spark or an electric arc, generally known as discharge. When a conducting material is subjected to a high-frequency electromagnetic field, the charges on the conductor move entirely to the conductor’s surface due to the low penetration depth. However, the charges on the material’s surface do not distribute themselves uniformly. Instead, the distribution depends on the detailed shape of the metals. At sharp edges, tips and submicroscopic irregularities, the surface charge density and the external electric field may reach very high values. When some charges accumulate enough kinetic energy, they will jump out of the material, resulting in the ionization of the surrounding medium, producing an electric discharge [62]. The conductive material in the microwave field plays a very important role in the discharge process by supplying electrons for initiating, sustaining and completing the discharge [25,62,94,95]. As a direct consequence of these discharges, considerable heat is produced, leading to the formation of high-temperature local hotspots. Wang and co-author reported the heat produced by discharge can melt the metal terminals (as shown in Figure 4) and the energy conversion efficiency from the electric energy to heat by microwave-metal discharge can amount to 20%–60% during the microwave irradiation process, as shown in Figure 5 [96,97]. One the other hand, at a microscopic level, these discharge are actually plasma [12]. An intensive generation of such hotspots and plasma is potential to affect the chemical reaction process and the composition of products, largely, and therefore may have significant implications for the processes involved.

Although the discharge phenomena triggered by microwave irradiation on metals may play an important role in various technological and scientific fields, the understanding of microwave-metal discharge is currently still insufficient. To date, only a few attempts have been made to investigate the physical nature of microwave-metal discharges. For example, Hu *et al.* [98] demonstrated that electrons escape from the metal terminals and produce an arc discharge in the microwave field when the induced potential exceeds the coulomb potential and there should be a critical length determining whether arc discharge caused by microwave irradiation on the metal wire could occur or not. Arc discharge can only occur when the metal wire is longer than the critical length. Wang *et al.* [96] investigated the influenced factors of microwave-metal discharge and demonstrated that the factors such as the microwave power, irradiation time, amount of metals, and atmosphere are all important variables that influence the discharge intensity and the overall heating effect. Sun *et al.* [97] investigated the coupling mechanisms related to the heating effect between wave absorption and metal discharge and pointed out that the wave absorption capacity of the surrounding medium exerted a critical influence on the stimulation and intensity of the discharge phenomena. Chen *et al.* [62] investigated the electric discharge phenomena in metal-solvent mixtures with particular emphasis on the discharges exhibited by different metals (Mg, Zn, Cu, Fe, Ni) of varying particle sizes and morphologies in organic solvents (e.g., benzene) at different electric field strengths. They pointed out that various factors, such as magnetron output power, amount, size, morphology and physical properties of the conductor or semiconductor influence the breakdown processes in a given solvent [62]. It should be pointed out that current studies are mainly limited to the disclosure of the influence factors and function rules of the microwave-metal discharge phenomena based on the experimental results. It does not unveil the microwave-metal discharge mechanism from a physical origin. More work is still eagerly awaited to unveil the nature of microwave-metal discharge mechanisms.

Despite the insufficient understanding of the physical nature of the discharge phenomena, the heating effect and plasma effect caused by microwave-metal discharges have received a growing amount of attention worldwide. Gasner *et al.* [99] showed that microwave pyrolysis of coal could be greatly promoted by inserting two paralleled copper wires into the coal sample, and concluded that a plasma might form between the two wires. Hussain [100] found the interaction of microwaves with iron led the iron’s temperature to rise up to 1100–1200 °C and even up to the melting point of iron. Kando *et al.* [101] carried out some research on the excitation of microwave discharge by an antenna to produce high intensity microwave discharge and found a plasma column emitting strong radiation was produced in a small gap between a couple of antennas and the plasma column sometimes changed to a curved one with the increase of the incident microwave power. Chen *et al.* [62] reported that audible and bright discharges accompanied by solvent decomposition and formation of considerable amounts of graphitized material were observed by adding metal particles (Mg, Zn, Cu) to liquid benzene under microwave irradiation. Since benzene is completely microwave transparent, the decomposition reaction is attributed to microwave-metal discharge, indicating microwave heating can be extended into more fields through the introduction of electric discharge (*i.e.*, promoting the chemical reactions in microwave transparent solvents). Ma [102] have studied the catalytic reforming effect of CH_4_ and CO_2_ to produce H_2_ and CO, found the conversion efficiency of CH_4_ and CO_2_ can reach 91.76% and 87.20% respectively, with the selectivity of both H_2_ and CO upgraded to approximate 80% by passing through microwave-metal discharge area.

Despite the large body of published work in the field of microwave-assisted pyrolysis processes, microwave-metal discharges have been innovatively applied in the material synthesis. For instance, microwave-metal discharge can be used to promote the insertion of Mg metal into carbon-halogen bonds to form Grignard reagent since the interaction between the microwave field and the Mg turnings can produce clearly visible electrostatic discharge phenomena (arcing) between the Mg particles, causing a distortion of the Mg surface which results in smaller and thus more reactive spherical Mg particles, and possibly also a removal of the passivating MgO/Mg(OH)_2_ layer present on the non-activated Mg turnings [103,104]. Additionally, microwave-metal discharge can also be used to promote the sintering of metallic particulate to achieve better heating efficiency and shorter processing times since micro-plasmas may generate during microwave heating of metals particles, and plasma ignition between particles may take place to enhance necking and heating processes [26].

On the basis of the research progress, it can be concluded that microwave-metal discharges are critically dependent on many factors, including the applied magnetron output power, number, size, morphology and surface conditions of the metals, dielectric loss tangent of the surrounding medium, and some other factors. The influence factors are not independent; they work together to shape the microwave-metal discharge phenomena. In general, discharges (including, corona, spark, and arc discharge) are prone to be stimulated when metals with sharp edges, tips or submicroscopic irregularities are subject to a microwave field. Accompanied with discharge, high-temperature hotspots and plasmas are two featured effects which can play an important role in promoting microwave heating processes and chemical reactions. Microwave-metal discharge could, therefore, play an important role in a variety of applications such as the microwave-assisted pyrolysis, material synthesis, pollutants removal, microwave-assisted formation of nanomaterials and many other fields.

## 4. Important Ways to Improve Microwave Energy Efficiency

Despite microwave heating technology has been applied in various fields due to its distinct advantages, the energy efficiency is an important issue determining whether it can be economically competitive with other technologies and applied in industrial practice for commercial purposes. Thus, it is of great significance to explore the novel strategies to improve the microwave energy utilization efficiency. Following are some important solutions based on the paper reviews and our previous investigations.

### 4.1. Specific Set of Reactor Dimension with Full Consideration of Penetration Depth, Microwave-Matter Interaction Mechanisms and Constructive Interference of Propagating Waves

**Figure 6:**
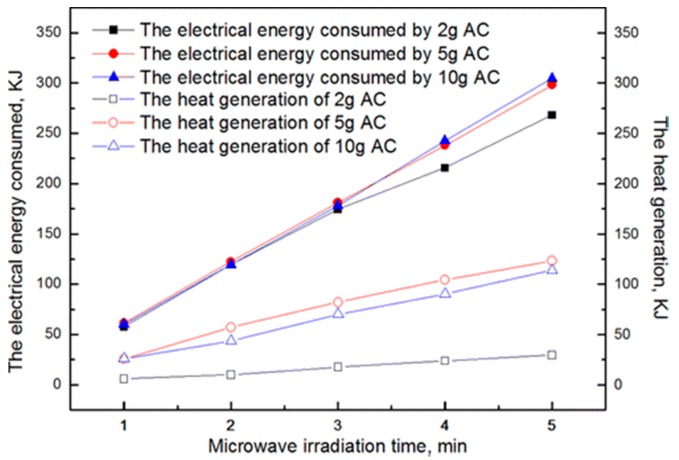
The heating effect of AC in microwave field at 700W [97]. Reprinted from (Industrial & Engineering Chemistry Research 2014, 53, 2042-2051) with permission from American Chemical Society.

Microwave heating typically occurs in the bulk of a reaction sample through penetration of themicrowaves. As is mentioned in Section 2.4, the overall heating efficiency is sometimes low, even though the material to be heated is a good microwave absorber owing to the shallow penetration depth of the microwaves. Works carried out by Sun and her co-authors indicate the heating uniformity and energy conversion ratio related to microwave heating of good microwave absorbers could be improved when they are mixed with some microwave transparent materials. For example, the overall heating effect of 10 g pure AC (AC is short for activated carbon) was inferior than the 5 g AC mixed with 25 g high-purity quartz sands (microwave transparent material) when they are irradiated in the same spherical volume (diameter is 40 mm) and microwave conditions, as is shown in Figure 6 [97]. As AC is a strong microwave absorber with a large dielectric loss [105], the power dissipation capacity is very strong, while the microwavepenetration depth is very low, only the outer layer of the activated carbon can be effectively heated by microwaves, while the inner layer is heated via heat transfer. When AC is diluted with quartz sands, the effective dielectric loss must be decreased, leading to decreased power dissipation capacity, while the wave-penetration depth is increased, consequently, it can be heated more uniformly by microwaves with possible improved energy conversion efficiency when compared with the pure AC [97]. Obviously, an optimal sample dimension with the consideration of the penetration depth plays an important role in an efficient heating process.

Here, in order to estimate the penetration depth, the importance of the measurements of complex dielectric permittivity (ε) and complex magnetic permeability (μ) should be highlighted since elucidation of these properties of each component and the bulk reaction mixture is essential in understanding the interaction of electromagnetic waves with a material and the penetration depth, and consequently would be helpful in the optimization of microwave thermal processes. Moreover, the lack of dielectric data in the microwave frequency range as a function of temperature is widely recognized as a limitation to better incorporate the material into its intended applications or explain some unclarified mechanisms. Thus, the measurement of temperature-dependent dielectric and magnetic parameters at microwave frequency has gained increasing importance and interest by both academia and industries since a wide variety of fields, such as material science, absorber development, biological research, *etc.*, need a better understanding of the materials they are working with to optimize process design [106,107,108,109]. Accurate measurements of these properties can provide scientists and engineers with valuable information to properly incorporate the material into its intended application for more solid designs or improved quality control or optimization of some processes.

In addition to the dielectric data, the interaction mechanisms also have a profound effect on the penetration depth since the E-field and H-field of microwaves interact very differently with matter, the heating behavior may vary a lot even for a given sample. Take the microwave heating of aqueous electrolyte solutions for example, both dielectric dipolar polarization losses and ionic conductive losses contribute to the E field heating, while the heating behavior in H field mainly attribute to the associated Joule heat of the current induced. Owing to the different interaction mechanisms, the penetration depth of E-filed and H-filed into a liquid/solution may vary a lot, resulting in different performances under separated E-field and H-field irradiation. For instance, with the increase of the electrolyte concentration, the heating rates of the aqueous electrolyte solutions(NaCl, KCl, CaCl_2_, *ect.*) were reported to exhibit a near-exponential growth under H-field irradiation, while the heating rates increased to a plateau at low electrolyte concentrations and remained nearly the same with further increase of electrolyte concentrations under E-field irradiation [42]. This remark difference is attributed to the different evolution of penetration depths. The penetration depth of the microwaves under E-field irradiation becomes shallower on increasing the electrolyte concentration. In contrast, the magnetic field heating is nearly independent of penetration depth because the solution does not absorb the magnetic irradiation and the alternating magnetic irradiation can penetrate the entire solution to induce ring currents everywhere, causing the solutions to be heated up [42]. Thus, a good understanding of the interaction mechanisms is another important precondition for choosing the suitable microwave heating mode and setting the sample dimensions correspondingly.

According to Equation (5), the microwave power absorption also depends on the electromagnetic field strength within a material. As mentioned in Section 3.1, the electromagnetic field strength can be modified based on the interferences of propagating waves. Constructive interference leads to resonances and causes a dramatic increase of power absorption while destructive interference suppresses power absorption. On one hand, the proper introduction of foreign support (e.g., metallic support or metallic-ceramic support) has been proven to be an effective way to strengthen the constructive interferences and power absorption in certain material with suitable sample dimensions [9,61,63,64,65,70]. It was reported that enhanced energy savings (as high as 60%) can be realized by the use of metal coated bounding surface at the other side of microwave incidence for reactors with 2L/λeff = 0.5n − 0.25, where n = 1, 2, 3…, 2L is the reactor dimension, and λeff is microwave wavelength within the reactor. The enhancement is found to be 2 and 1.5 times at 2L/λeff = 0.25 and 0.75, respectively [70]. On the other hand, destructive interferences can be avoided by suitable alteration of the interference pattern via varying sample dimensions and material dielectric properties [110]. Basak reported substantial enhancement of microwave power absorption with optimal thermal runaway can be achieved via splitting a large continuous sample into two discrete parts which were separated by air layer and the thickness of air layer can influence the intensities of incident and reflected electric field in each discrete sample layer to form constructive interference [111].

To conclude, for microwave heating of a given material, the sample dimensions should be set with the full consideration of the penetration depths, interaction mechanisms and interferences of the propagating waves in order to achieve an energy efficient process. The measurements of complex dielectric and magnetic data plays an important role in estimating the penetration depth, interaction mechanisms and interference patterns of the propagating waves within the material.

### 4.2. Microwave Energy Conversion Enhanced by Microwave Absorber

Microwave heating is a good alternative for carrying out many chemical or physical processes such as catalytic heterogeneous reactions, disposal of hazardous waste (e.g., electronic waste) and pyrolysis of various organic wastes (e.g., biomass, sludge, oil shale, plastic waste, *etc.*). In some conditions, the materials to be processed are poor receptors of microwave energy, so they cannot be heated up to the high temperatures that usually required for reaction. With the aid of some good microwave absorbers, microwave-induced disposal is possible. In the past several decades, microwave absorbers (such as SiC, carbon materials, metal oxides, *etc.*) have been widely used in facilitating the microwave treatment of some poor receptor materials, resulting in a considerable improvement in the energy and processing efficiencies.

Microwave absorbers can be used as susceptor or substrate to facilitate the heating of substance that cannot be heated effectively and efficiently by microwaves via heat transfer since the absorber can perfectly couple with the incident microwaves to heat itself efficiently. This approach is also known as hybrid heating. In 1990s, Wicks, *et al.* [112] disclosed the utilization of microwave absorbers as a susceptor to recover metals from waste in their patents. Ripley [71] also reported the combination of ceramic crucible and thermally insulating casket can be a better way to treat poor wave-absorption materials efficiently by microwave irradition. The ceramic crucible used to hold the treated objects strongly absorbs microwave energy to heat itself uniformly to the desired temperature, and then heat the treated samples via radiation, conduction and convention. The thermally insulating casket, which is microwave transparent, increases the energy efficiency of the system by trapping the heat generated in the crucible. It should be pointed out that the hybrid approach can also partially hide the benefits deriving from the direct microwave–matter interaction, such as rapid, volumetric and selective heating characteristics.

Despite the indirect use of absorbers as susceptor/substrate, microwave disposal efficiency can be greatly improved if microwave absorber is directly added into the treated materials. Extensive studies show carbon materials are usually preferred absorbers to improve the energy conversion efficiency, not only because they can couple with microwaves efficiently to heat themselves rapidly, but also because they are inexpensive, easily available in different textures, sizes, forms, *etc.* Moreover, it will not introduce any extra inorganic component to the solid residues in the pyrolysis processes of various organic wastes. The carbonaceous residue itself which is obtained from the pyrolysis of the organic materials can also be used as an excellent microwave absorber [5,11,12].

Additionally, some microwave absorbers can also be used as a support/carrier for some catalyst to carry out microwave catalytic reactions. For example, metallic catalysts loaded on activated carbon have been widely used in an environmental clean-up and organic synthesis. It was reported that considerable improvements in the yield of the reactions has been realized in the fields of NOx reduction [113,114], SO_2_ reduction [115], catalytic CH_4_ decompositionfor H_2_ production [116,117] and CO_2_ reforming of CH_4_ (dryreforming) [118,119], *etc.*

In conclusion, the adoption of microwave absorbers to facilitate microwave heating and processing processes has been successfully applied in practice for many years and could be implemented further in more applications. With the developed understanding of microwave-matter interaction mechanisms, it is possible to tailor the introduction of microwave absorber for a given process and then enhance microwave energy and processing efficiencies to a new level.

### 4.3. Improved Microwave Processing and Energy Efficiency Owing to Microwave-Induced Discharge Effect and/or the Coupled Effect of Wave Absorption and Discharge/Plasma

Despite the heating mechanisms, another unique mechanism deserves full consideration is the microwave-induced discharge effect. As is mentioned above in Section 3.3, high-temperature hotspot and plasma effect are two important phenomena associated with discharge, which can exert a significant effect in promoting microwave heating processes and chemical reactions. In our previous research on microwave-induced pyrolysis of electronic waste, we found that microwave-metal discharges occurred immediately at the onset of microwave irradiation, leading to several local hot spots and a great promotion of the pyrolysis processes. Owing to the microwave-metal discharges, it is possible to decrease the activation energy related to the pyrolysis process and increase the yield of H_2_ [39]. The previous and ongoing studies in our laboratory have proven that the hotspot effect is associated with considerable energy conversion (from microwave energy to heat) [38,39,96,97]. As is well acknowledged, the plasma that contains highly active species such as electrons, ions and radicals can significantly enhance the reaction rate. Plasma usually plays a catalytic role even though there is no catalyst in the reaction. Microwave-induced discharge is one important technique used to obtain a non-equilibrium plasma, even under atmospheric pressure, at which the electron temperature is approximately 4000–6000K, while the heavy particle temperature is around 2000 K [120]. Take the steam reforming of hydrocarbons for example, when steam is used as the plasma supporting gas in microwave discharge, radicals such as H·, ·OH and ·O, as well as high-energy electrons are generated. Moreover, since both reductive and oxidative conditions are provided in the plasma, it is concluded that steam plasma is effective for various treatments of materials [120]. Due to its versatile capabilities, microwave-induced plasmas has attracted a lot of attentions and applications, including hydrogen or syn-gas production via microwave plasma reforming of various hydrocarbons such as methane, iso-octane, gasoline, *etc.* [120,121,122], material synthesis [123], contamination removal from gas stream [73], and more.

It should be pointed out that the discharge phenomenon in the microwave field is usually accompanied by the wave absorption processes. When the wave absorption processes and the discharge/plasma phenomena coexist under microwave irradiation, both of them will dissipate the microwave energy, involving an energy conversion process. From the perspective of energy conservative, there is an optimal energy conversion efficiency due to their coupled effect. In our previous work, we investigated the coupling mechanisms related to the heating effect between wave absorption and metal induced discharges in microwave heating processes. We found that he coupling mechanisms for direct microwave absorption and microwave-metal discharges varied from collaborative to competitive with the increase of microwave absorber since the stimulation of microwave-metal discharges becomes harder when the amount of microwave absorber is relative large. An optimal energy conversion efficiency can be realized by the collaborative heating effect of wave absorption and discharges [97].

In conclusion, microwave-induced discharges or the collaborative effect of wave absorption and microwave-induced discharges can be exploited to facilitate the microwave heating/processing in a variety of applications. With the assistance of microwave induced electric discharges in various microwave heating scenarios, the microwave disposal processes and their efficiency can be optimized rationally, perhaps enhancing the energy efficiency to a new level.

### 4.4. Numerical Modeling Plays an Important Role in the Optimization of Microwave Thermal Process

The application of microwaves for thermal processing of materials is an interdisciplinary subject since the heating by microwave radiation constitutes a highly coupled nonlinear problem which combines several fields of physics and materials science. In general, the description of a microwave heating process typically comprises an analysis of the distribution of microwave electromagnetic field in the material and the absorption of the electromagnetic energy therein [124]. Among the wide number of description of microwave heating process in the scientific literature, a more general one considered by the author was proposed by Rybakov [124]: The distribution of the electromagnetic field within the object generally depends on (1) the effective dielectric and magnetic properties of the material that depend on temperature and structural variables such as porosity; (2) the dimensions and properties of the applicator in which the processing is undertaken; (3) the matching conditions between the microwave source, transmission line, and applicator, *etc.* Similarly, the distribution of temperature within the heated object is determined by: (1) the distribution of electromagnetic field within the material; (2) the effective absorption properties, heat capacity, and thermal conductivity of the material (depending on temperature and porosity); (3) the conditions of heat removal that may involve properties of thermal insulation, emissivity of the material, convection, *etc.* Owing to the highly coupled nonlinear characteristics and many interdependent variables, the experimental optimization of microwave processing regimes is labor intensive and time consuming. Moreover, microwave-matter interaction sometimes gives rise to new and unexpected physical behavior, *i.e.*, the best known appearance of “hot spots”, which are localized areas of high temperature that may develop during the microwave radiation. Hot spots are undesirable for some processes (*i.e.*, sintering of ceramics) since it leads to product damage whereas in some other processes (*i.e.*, microwave assisted pyrolysis) hot spots are desirable to quicken the process and improve the energy efficiency. Hence, it is necessary to predict the condition under which hot spots arise, so that their occurrence can be either avoided or utilized. However, it is usually difficult to detect the hotspots by the direct measurement in temporal and spatial scales. Numerical modeling from which insight might be gleaned into an inherently complex physical process plays an important role in predicting the heating behavior and occurrence of such phenomena.

In the past few decades, the increasing interest in industrial applications of microwave heating, such as in drying, melting, sintering, and smelting has prompted a considerable amount of research that aims to formulate numerical models capable of accurately modeling the processes. The development of mathematical modeling of microwave heating has been widely reviewed by Hill [125], Ayappa [126], Rybakov [124] and Chandrasekaran [15]. Numerical modeling plays an important role in proper designing of the microwave cavities for various applications [127], predicting microwave heating rates and associated temperature patterns [128], calculating the power absorption and energy conversion ratio [129], facilitating the interpretation of some uncommon mechanisms such as microwave heating of metal powders [3], *etc.* Based on its powerful functions, numerical modeling doubtlessly plays an important role in optimizing the heating strategies to improve the microwave energy utilization efficiency.

When a foreign object is introduced into microwave cavity, the electromagnetic field intensity can be highly distorted compared with the fields within an empty cavity [128]. Even for the same object, the interference on the electromagnetic field distribution is different when it is put in different location in the microwave cavity. Moreover, as we mentioned above, the object dimensions as well as the reactor configuration (with or without foreign support) also play a profound effect on the interference patterns [63,70,110,111]. Spatial maxima and minima of power absorption or heat generation may form within the material, owing to the constructive and destructive interferences of the propagating waves, respectively. For a give material, numerical modeling can facilitate in making a preliminary estimate on the interference patterns as a function of sample location in cavity, sample dimension, material dielectric properties as well as the reactor configuration (with or without foreign support), as a consequence, providing a useful guideline to minimize the destructive interferences and maximize the heating rate with optimal thermal runaway. To resolve the electric and magnetic field distributions within the cavity at a particular frequency, the interactions between all the dielectric materials and applicator structure must be quantified. The exact geometry of the cavity is required, as well as the relative three-dimensional position of all materials within it; the dielectric and magnetic properties of all materials as well as their dependence with temperature are also required. The change in the field distribution must also be considered over time, as the dielectric properties change with temperature. To date, some commercial software (*i.e.*, COMSOL multiphysics) has been maturely developed to obtain a spatial electric field distribution within the cavity, as well as the temperature profile of the heated sample, and calculate the power absorption or energy conversion efficiency, for the specific sample and experimental conditions used.

With the assistance of numerical modeling, the sample dimensions/disposal scale can be optimized for a given material at a certain microwave frequency to achieve optimal heating rates and energy utilization efficiency. Moreover, since the penetration depth increase with the decrease of microwave frequency for a given material, despite the commonly used 2.45 GHz, the heating process at other operation frequencies can be modeled to finding the best coupling between microwaves and certain samples; for example, for some extra large samples, some lower frequency (e.g., 915 MHz) may be more energy efficient, owing to the relatively large penetration depth and uniform heating. In addition, the heating process of some low-loss materials aided by microwave absorbers or susceptors or microwave-metal discharges can be numerical modeled to optimize the heating strategies and energy conversion efficiency by incorporating the temperature-dependent dielectric and magnetic properties of each component and the bulk reaction mixture into the numerical modeling with proper boundaries. For instance, Shukla *et al.* [130] conducted numerical modeling to investigate the influences of sample dimension (cylinders of varying radii), physical properties, and susceptor on the heating behavior of samples in microwaves and found the efficacy of microwave heating depend on the sample size and its thermal conductivity. More recently, Klinbun and Rattanadecho [131] investigated the influences of physical parameters, e.g., microwave power level, placement of sample inside the waveguide, and volume of sample, on the distribution of electric field, temperature profile and velocity field for microwave heating of water layer in a rectangular waveguide. Cherbański and Rudniak [132] conducted numerical modeling to investigate microwave induced natural convection in water, and found that a deviation of only 5 MHz from the nominal frequency (2.45 GHz) changes the absorbed microwave power in water by about 20%.

It should be pointed out that although the numerical modeling of the microwave process has been developed for several decades, there are still some challenges and unsolved problems, which are mainly due to the insufficient understanding of microwave-matter interaction mechanisms; for example, the numerical modeling of microwave-metal discharge process is still lacking in literature owing to the limited understanding of the physical nature of the microwave-metal discharge phenomena. With the developed understanding of microwave-matter interaction mechanisms, it is possible to boost the utilization of numerical modeling for the optimization of microwave processes in more fields in the near future.

## 5. Concluding Remarks and Future Scope

Microwave heating is commonly known as dielectric heating and its application is usually limited to microwave electronic field heating. The information presented in this review shows that microwave magnetic field heating cannot be ignored and contributes greatly to microwave heating of some aqueous electrolyte solutions, magnetic dielectric materials and conductive powder materials, *etc.* In order to present a sound interpretation of the microwave–matter interaction mechanisms, microwave E-field and H-field heating fundamentals are reviewed and discussed in a comprehensive manner in this paper. Also, the special effect of microwave interaction with metal-based materials is explained in detail, which may open up several promising ways to induce microwave resonance, eddy current and plasma effect.

Although many research studies have been conducted to examine the advantages of using microwaves instead of traditional heating techniques, there are limited industrial applications of microwave heating technology due to the fact that microwave energy is expensive in terms of capital cost and energy conversion efficiency, resulting in microwave technology being less economically competitive than using traditional techniques. Consequently, important strategies to optimize the energy process involved to achieve the best results with minimum energy consumption are required. Herein, four important strategies to enhance the microwave energy conversion efficiency are proposed and discussed.

It can be said that microwave technology has many unique advantages that are waiting for greater exploitation and more applications. With a more developed understanding of the physical nature of the coupling mechanisms between microwaves and matters, it is promising to extend the utilization of microwave technology for more commercial or scientific purposes.

## Figures and Tables

**Table 1:** Penetration depth of Mg, Zn, Cu, Fe and Ni at 2.45 GHz [62].

Metals	Mg	Zn	Cu	Fe	Ni
Penetration depth, μm	2.2	3.2	2.7	1.3	2.5
